# Cystic fibrosis and inflammatory bowel disease: parallels in gut physiology and microbiota

**DOI:** 10.1128/jb.00167-25

**Published:** 2025-08-18

**Authors:** Kaitlyn E. Barrack, George A. O'Toole

**Affiliations:** 1 Department of Microbiology and Immunology, Geisel School of Medicine at Dartmouth 12285, Hanover, New Hampshire, USA; University of Virginia School of Medicine, Charlottesville, Virginia, USA

**Keywords:** cystic fibrosis, inflammatory bowel disease, microbiome, dysbiosis

## Abstract

Cystic fibrosis (CF) and inflammatory bowel disease (IBD) represent distinct pathologies with unique genetic underpinnings; yet, they share remarkable similarities in gut microbiota dysbiosis and intestinal physiology. This review comprehensively examines the parallels and differences between these conditions, focusing on microbial signatures, inflammatory markers, and physiological features. Both diseases exhibit increased levels of Proteobacteria, decreased anaerobic short-chain fatty acid producers, and altered intestinal metabolic profiles. Common physiological characteristics include intestinal inflammation with elevated inflammatory markers (calprotectin, S100A12, lactoferrin), lower intestinal pH, and similar bile acid dysregulation patterns. However, key differences emerge in mucus characteristics, disease onset timing, and current treatment approaches. The gut microbiota plays a crucial role in both conditions, with shared signatures of dysbiosis suggesting similar intestinal environmental shifts and potential common therapeutic targets. Recent advances in CFTR modulator therapy have shown promising effects on the CF gut microbiome, while IBD treatments demonstrate variable efficacy. Understanding these similarities and differences is crucial for developing targeted therapies that could benefit both populations. This review highlights the complex interplay between host genetics, environmental factors, and the gut microbiota, emphasizing the need for further research to disentangle these relationships. We also discuss how the information provided here can be used to build and validate *in vitro* models to study the dysbiotic microbial communities and their causes in these diseases, to develop more effective treatments.

## INTRODUCTION

The human body harbors 10–100 trillion microbial cells (1, 2), encompassing bacteria, fungi, and viruses. These microbes reside across many body sites, including the skin, mouth, nose, reproductive tract, and intestine (3). The vast majority of microbial cells in and on the human body reside in the gastrointestinal (GI) tract, specifically the colon (3, 4). The gut microbiome contributes myriad benefits to the function of the intestine, including digestion, absorption, metabolism, barrier integrity, immune system training, vitamin production, and protection against pathogen colonization and infection (1, 3). The composition and function of these microbes differ at each anatomic site, thereby defining the “microbiome” of the given gut region. Imbalances in the microbial community, known as dysbiosis, are associated with various diseases, such as inflammatory bowel disease (IBD), cystic fibrosis (CF), obesity, diabetes, allergy, and neurological disorders (4–9). Dysbiosis and disease manifestations are thought to be bidirectional, whereby alterations in microbiota can either precede a disease-related event or be a result of said event; this bidirectionality is frequently observed in the context of respiratory diseases (10). Thus, it is often difficult to identify the direct implications of the gut microbiome and its role in complex disease states (11).

Ample evidence suggests that microbiomes have functional effects across distant body sites (9, 10), proposing axes of communication or crosstalk between organs facilitated by microbes and/or their metabolites. This observation is unsurprising considering the intricate microbial networks that are observed in natural environments (i.e., soil, aquatic environments, mycorrhizal networks) (12–14). Scientists have found links between the gut microbiome and the brain, lung, liver, kidney, pancreas, and the reproductive and cardiovascular systems (15–22), largely driven by the metabolites and products of microbial metabolism originating in the intestines. In the context of CF, a genetic disease in which the secretion of chloride and bicarbonate is impaired across membranes, impacting multiorgan mucosal layers, several such cross-organ axes have been investigated. Our group has primarily focused on the gut-lung axis (10), where we observe a correlation with high alpha diversity of the gut microbiome and better lung health outcomes (e.g., longer time to first pulmonary exacerbation) (16, 23). In addition, gut microbiota composition correlates with linear growth in a children’s CF cohort (24). When investigated further, a specific taxon, *Bacteroides*, resident in the CF gut at lower levels than in healthy controls, is involved in mediating systemic inflammation via the secretion of the short-chain fatty acid (SCFA) propionate (25, 26). These data support the idea that the gut microbiota and its products modulate processes in distal organs; this connection can be disrupted by an imbalanced gut microbiota.

Interestingly, there exists a complex interplay for diseases impacting multiple organ sites. For example, nearly 40% of IBD patients are diagnosed with depression during their lives (27, 28), indicative of a strong correlation between intestinal and neurological disorders. While the mechanism of this interaction is not yet known, hypotheses focus on the involvement of gut inflammation and the metabolism of tryptophan, an amino acid and precursor to serotonin (15). Therefore, proper microbial structure and function in the gastrointestinal (GI) tract are critical for whole-body function and well-being.

Here, we aim to comprehensively review the observed gut microbial dysbiosis across diseases, with a major focus on cystic fibrosis and IBD, and draw comparisons with reported physiological and microbiological features. While the etiology of each condition is distinct, the gut microbiomes often adopt similar signatures, suggesting the potential for shared disease mechanism and/or therapeutic approaches. By comparing each disease’s intestinal features, both microbiological and physiological, we aim to pinpoint features with therapeutic potential across disease states. Furthermore, understanding the altered physiology of these two disease states will allow us to better model these conditions in the lab with the goal of probing the relation between the altered intestinal environment and the associated intestinal microbial dysbiosis.

## GUT MICROBIOME DYSBIOSIS AND ALTERED METABOLITES ASSOCIATED WITH CYSTIC FIBROSIS

The gut microbiome of persons with CF (pwCF) is altered starting in early age (24, 25, 29, 30), and this imbalance persists later into adulthood (31–33). As early as 6 weeks of age, certain taxa, including those in the Bacteroidaceae family, specifically *Bacteroides*, are significantly depleted in children with CF (cwCF), relative to healthy age-matched controls (25). Within the first year of life, alpha diversity measures (Shannon Diversity Index) are significantly lower in CF stool microbiomes compared to healthy controls (24, 25). In CF, at the phylum level, Proteobacteria are increased, Bacteroidetes and Verrucomicrobiota are decreased, and Firmicutes and Actinobacteria levels are variable, depending on the study (10, 24, 31, 34–40). Key genera contributing to these alterations include *Escherichia*, specifically *E. coli* (Proteobacteria) (10, 24, 25, 34, 41), *Bacteroides, *specifically *B. vulgatus* (Bacteroidetes) (24, 25, 31, 34, 42, 43), *Akkermansia* (Verrucomicrobiota) (22, 34, 44), *Clostridium* (Firmicutes) (31, 37, 38, 44), *Blautia* (Firmicutes) (31, 38), and *Bifidobacterium* (Actinobacteria) (31, 36). In general, anaerobic taxa that are involved in SCFA production are decreased in CF stool communities, including *Faecalibacterium, Bacteroides, Akkermansia, Roseburia, Odoribacter,* and *Eubacterium* (10, 22). Bacterial members of the health-associated *Clostridium* cluster XIVa are also decreased in the CF fecal microbiota (45). Conversely, there is an observed enrichment of genera associated with pro-inflammatory conditions, including *Escherichia, Streptococcus, Staphylococcus, Veillonella, Clostridium,* and *Enterococcus* (10). For a comprehensive summary of the changes in gut microbiota in CF, please see our recent comprehensive review (10).

Beta diversity analyses comparing CF and non-CF stool microbiomes show differences in these communities across multiple forms of multivariate statistical approaches (24, 25, 29). Of note, CF stool microbiomes are distinguished by genotype with a higher effect size as age increases (24), suggesting a delay in maturation and further differentiation from non-CF stool microbiomes throughout childhood. Across different beta diversity measures (using both Bray-Curtis [24] and unweighted UniFrac [29]), clustering by genotype is largely driven by deviation in specific taxa: *Escherichia* and *Bifidobacterium*, with some contribution by *Ruminococcus* (29). *Bifidobacterium* is largely negatively associated with age in healthy cohorts; an enrichment of this genus in CF suggests a “younger” microbiome relative to non-CF cohorts. In fact, using regularized random forest models, two separate studies have shown that CF gut microbiome samples from children are delayed in maturation relative to non-CF age-matched samples (24, 29). CF stool microbiomes are significantly delayed relative to healthy controls beginning around 6 months of age (24), and this delay persists out to 3 years of age, although the rate of delay slows around 18 months (29). Further studies are needed to confirm the long-lasting consequences of this lack of typical developmental maturation and whether this delay is preventable through microbial or nutritional interventions (i.e., probiotics, prebiotics).

Not only do certain genera significantly shift in CF gut microbiomes, but these genera appear to drive community-wide differences within genotypes. More specifically, when CF microbiomes are compared to each other, in the absence of non-CF comparators, one study observed two distinct clusters following unweighted UniFrac beta diversity analysis (29). These clusters were largely driven by the presence or absence of *Bacteroides*, with all other tested covariates (breastfeeding, fecal fat, proton pump inhibitor usage, antibiotic treatment, and fecal calprotectin levels) proving non-significant in this differentiation. These data suggest that differences in beta diversity can be driven by the presence (and inferred metabolic activity) of a single genus—*Bacteroides* in this case. As observed in this study, whole microbiomes can shift independent of other clinical parameters, highlighting the dominant role of *Bacteroides* in CF gut microbiomes and its potential contribution to the development of dysbiosis.

With the consistent reduction in SCFA-producing microbes in CF microbiomes, several studies have investigated the abundance of these metabolites in stool samples using metabolomic approaches (33, 35, 37, 46). In children’s cohorts, there is a consistent depletion of butyrate and propionate, as well as certain intermediates of these metabolites (35, 37). Acetate is also depleted in one CF cohort, relative to healthy controls (37). In adult cohorts, all tested branched-SCFA trend lower in CF, with valerate and hexanoate reaching significance (33). As an alternative to directly quantifying these metabolites via H-NMR or gas chromatography coupled with mass spectrometry, other groups have inferred SCFA metabolism via -omics approaches. Manor et al. utilized metagenomics to quantify microbial pathway abundances in CF and non-CF children’s gut microbiomes (34). Here, an enrichment in fatty acid degradation microbial KEGG pathways and a depletion of fatty acid biosynthesis microbial KEGG pathways are observed in the CF cohort, suggestive of increased breakdown combined with decreased production of these beneficial metabolites. Of note, these catabolism modules have a significant positive correlation with fecal fat content and fecal calprotectin levels, a measure of neutrophil activity and proxy for inflammation (34). Alternatively, through proteomics of stool from cwCF compared to stool of their non-CF siblings, Debyser et al. showed that two microbial proteins involved in butyrate production originating from *F. prausnitzii,* 3-hydroxybutyryl-CoA dehydrogenase and acetyl-CoA acetyltransferase, are decreased in CF (47), consistent with the observation that SCFA producers and their functional proteins are decreased in CF microbiomes.

In addition to a reduction in SCFA producers, an observed decrease in sulfate-reducing bacteria (SRB) and archaea is reported in adult CF fecal samples (48). Among these samples, *Methanobrevibacter* species were the predominant members contributing to the shift. It is hypothesized that disease-related changes in the CF intestinal environment interfere with the ability of these organisms to survive. The reduction in SRB may be partially attributed to the lesser availability of their preferred electron donors: SCFAs, proteins, and molecular hydrogen (48). Alternatively, altered pH gradients can also disrupt the ability for SRB and methanogens to establish in the colon (49). The availability of these nutrients, in the context of energy usage and electron shuttling, as well as pH, has not been extensively studied in the CF intestine. Finally, as described below, there is also evidence for increased oxygen in the CF gut, perhaps impeding the growth of these organisms.

It is thought that reductions in microbiota-dependent metabolites, such as SCFA, are driven partially by a low-density microbiota state (29, 37, 50), in which there is a lower absolute abundance of microbial cells relative to a control. Other signatures of a low-density microbiota state include increased relative abundance of oral-derived bacteria and higher levels of fungi (29, 51). First, numerous studies have shown that there is an enrichment of oral-derived bacteria in the CF gut microbiome, including *Staphylococcus* (29, 40, 52), *Pseudomonas* (40, 52), *Streptococcus* (16, 23, 38), and *Veillonella* (16, 23). There are two hypotheses as to why there may exist an increased relative abundance of oral bacteria in the gut microbiota: (i) the expansion hypothesis, where oral bacteria invade the gut ecosystem and expand and (ii) the marker hypothesis, where oral bacteria transit through the gut and their relative increase marks the depletion of other gut bacteria (51). Evidence in the context of CF suggests no difference in absolute abundance, or microbial load, in CF microbiota (31), supporting the marker hypothesis rather than the expansion hypothesis. Second, while the gut mycobiota has not been extensively explored in CF, there is evidence suggesting an increase in fungal prevalence in CF cohorts compared to non-CF cohorts (29). Specifically, there is increased detection of *Saccharomyces* and *Candida* in CF stool; both genera are associated with inflammatory bowel conditions (29, 53, 54). Overall, while absolute abundance quantification methods like qPCR have not demonstrated differences in microbial load in CF gut microbiota, there are other indicators that suggest a low-density microbiota: (i) a reduction in microbiota-derived metabolites, such as SCFA, and an enrichment in (ii) oral microbes and (iii) fungal species.

## GUT MICROBIOME DYSBIOSIS ASSOCIATED WITH IBD: CROHN’S DISEASE AND ULCERATIVE COLITIS

Inflammatory bowel disease (IBD) is a group of conditions that are associated with inflammation in the GI tract. The two main types of IBD are Crohn’s disease (CD) and ulcerative colitis (UC), which share similarities in symptomology yet manifest in different ways. Symptoms of both CD and UC include diarrhea, abdominal pain, rectal bleeding, and weight loss (55). Vitamin and mineral deficiencies are common in CD and UC. CD can manifest along any region of the GI tract, from the mouth to the anus, and can affect entire layers of the intestine. By contrast, UC affects the mucosal layer of the colon, rectum, and anus, leaving the small intestine largely unaffected. CD often has an early onset, between the ages of 15 and 35 years (55); similarly, in about 25% of cases, UC is diagnosed before the age of 18 years (56).

The causes of IBD remain largely unknown, although there is ample evidence pointing to the roles of genetic and environmental factors. Two frontline factors that drive IBD onset involve (i) the disruption of the mucosa and its consequent heightened immunological responses to the resident gut microbiota, and (ii) the contribution and exacerbation of the imbalance of the gut microbiota on pathology driven by the immune system (55, 57, 58). Intestinal microbial dysbiosis has been observed in persons with IBD and is largely characterized by a decrease in commensal bacteria diversity, with microbial members largely belonging to the Firmicutes and Verrucomicrobiota phyla (59–63). Taxa in the Firmicutes phylum that are consistently depleted in IBD intestinal samples are *Faecalibacterium prausnitzii* (62, 64–69), *Dorea* spp. (68), *Anaerostipes* spp. (68), *Butyricicoccus* spp. (68). Taxa in Verrucomicrobiota, specifically *Akkermansia muciniphila*, are reportedly decreased in both UC and CD stool (63, 70–72). Members of Actinobacteria are also decreased in both conditions, including *Bifidobacterium* (66, 73) and Coriobacteriaceae (68). While reports of Bacteroidetes abundances are variable in CD and UC, several studies have shown an increase in fecal levels of enterotoxigenic *Bacteroides fragilis—*strains that encode *B. fragilis* toxin (BFT) that can disrupt epithelial cell function and promote inflammation (74–76).

In both CD and UC, there is also a reported enrichment in the phylum Proteobacteria, including bacterial members of the Enterobacteriaceae family, specifically *Klebsiella* species, *Salmonella* species, and *E. coli* (60, 61, 77–85). *E. coli* strains that encode the genotoxin colibactin, also known as *pks+* (polyketide synthase positive) strains, are also enriched in inflammatory conditions (86–88); this toxic activity leads to double-stranded DNA breaks in epithelial cells, increasing the risk of colorectal cancer (89). Other members of the Proteobacteria phylum are enriched in IBD, such as *Campylobacter* and *Pseudomonas* (90–93). Pathogen abundance is associated with most IBD cases. The four well-studied pathogens are *Mycobacterium avium paratuberculosis* (*MAP*) (94, 95), adherent-invasive *E. coli* (AIEC) (96–98), *Enterococcus faecalis* (65, 78), and *Clostridium difficile* (82, 99, 100).

Another microbial signature associated with IBD includes an increase in sulfate-reducing bacteria (SRB) (62), including those in the families Desulfovibrionaceae (*Bilophila, Desulfovibrio*) and Desulfuromonadaceae (*Desulfuromonas*) (101) and Coriobacteriaceae (*Atopobium*) (102–104). SRBs are characterized by the metabolism of sulfate, an alternative electron acceptor reported to be increased in inflammatory diseases (105), into hydrogen sulfide, a toxic molecule that impacts butyrate oxidation, microbial viability, and phagocytosis (59, 62). Through the process of sulfate metabolism and subsequent oxidation of butyrate, coupled with the reduction in SCFA producers, SCFAs are depleted in the IBD gut, leading to lesser expression of tight junction proteins in the epithelial cells, thus increased gut permeability. With a leaky gut and excess hydrogen sulfide in the intestinal lumen, microbes and their products can translocate across the epithelia and evade elimination via phagocytosis. Such invading pathogens risk systemic inflammation while inducing a pro-inflammatory response via excessive Toll-like receptor stimulation (59), further compounding existing inflammation.

For CD, there is a consistent depletion of *Dialister invisus* (66), *Ruminococcus* spp. (65, 106), members of the Clostridium cluster XIVa (*Clostridium coccoides* group) (66), and members of Eubacteriaceae (65). These microbes are known to be involved in producing SCFAs (85). Alternatively, other taxa known to produce SCFA, specifically those in the Bacteroidetes phylum (*Bacteroides vulgatus* and *Bacteroides fragilis*), have been reported to be increased in some cases of CD (107). Since members of the genus *Bacteroides* are mucin degraders, it is hypothesized that an enrichment of these microbes may be contributing to damage to the protective mucosal layer (63, 108). However, *Bacteroides* spp. abundances are inconsistent across studies, with some reporting decreases in both *B. fragilis* (109–111) and *B. vulgatus* (112, 113). Considering the multifaceted role of these microbes in mucin degradation, polysaccharide A, and SCFA production, yet some with the capability to encode toxins (e.g., *B. fragilis* toxin), it is likely that the intestinal niche, physiology, and microbial ecology directly impact their abundances and functions. Taken together, while the suite of specific taxa altered in CF and CD may be different, there are conserved microbial functions that are similarly shifted, namely the reduction in SCFA production.

In UC cohorts, there is a similar decrease in Firmicutes members, including the *Clostridium* cluster XIVa (114). In addition, epithelium-associated levels of Bifidobacteriaceae are lower in active and quiescent UC, compared to controls (115). Counts of members of the Bacteroidetes phylum are variable: both increased (107, 114, 116, 117) and decreased (118, 119) detection in UC have been reported. Of note, *B. vulgatus* is one of the species within Bacteroidetes that is depleted in UC intestinal samples (119), showing some concordance with CD (65, 113) and CF (29, 34).

Unlike bacteria, the diversity of archaea appears to be higher in IBD patients compared to healthy controls. In healthy cohorts, a single predominant taxon is often present: *Methanobrevibacter*. In adult IBD cohorts, non-methanogenic archaea, such as Halobacteria, Nitrososphaerales, and Thermococcales, reach considerable levels (120–122). However, the prevalence and abundance of methanogens in the gut, specifically *Methanobrevibacter smithii*, decreases in IBD, particularly in those with UC (123, 124). This trend is also observed in pediatric UC cohorts (123). Interestingly, this decline in methane-producing archaea is not observed in young CD patients (123, 125, 126). Only recently did a study show that archaeal composition eventually shifts after long-term CD, resembling a profile more similar to UC (120). Together, these findings suggest that severe inflammation likely impacts the survival of methanogens, specifically in the colon, where there is typically a high archaeal load in healthy cohorts (53, 127).

The mycobiota, or fungal microbiota, associated with IBD is also altered (126–129). Beta diversity analyses performed on fungal genera demonstrate clustering by disease phenotype, whereby samples from patients with IBD in flare cluster separately from healthy samples (127). Of note, this separation is more pronounced when comparing samples from patients with UC in flare, compared to healthy samples. In addition, bacterial alpha diversity is decreased in both CD and UC, but fungal alpha diversity is decreased only in UC (127). These data suggest that, in acute states of inflammation, the fungal community in the intestinal tract shifts relative to healthy states, and this trend is exacerbated in UC. Specific fungal taxa driving these changes include decreases in members of the Ascomycota phylum, including *Saccharomyces* and *Malassezia*, and increases in members of the Basidiomycota phylum (127). Of note, *S. cerevisiae* has been shown to reduce colitis induced by AIEC in mice with ileal CD (130). Interestingly, *Candida albicans* is increased in CD (131), and absolute abundances determined by qPCR are significantly increased in IBD flares compared to IBD in remission (127). These shifts in fungal abundance in inflamed states can direct altered relationships with bacteria. Specifically, there is an observed positive correlation with *Saccharomyces* and *Bifidobacterium, Blautia, Roseburia,* and *Ruminococcus*, all of which are decreased in IBD (127). These findings suggest the presence of a complex inter-kingdom network that is directly impacted by inflammation, a phenomenon that is also observed in the CF airway (132, 133) and likely the CF intestine, due to the gut-lung axis (10), although this aspect of the disease has been less explored. Only one study of children’s stool microbiomes has investigated differences in the gut mycobiota (29). Here, the authors report an increased prevalence, or frequency of detection, of *Candida* and *Saccharomyces* in CF compared to non-CF. While investigation of the intestinal mycobiota is limited in CF cohorts (29), early results suggest shared fungal signatures with IBD cohorts, which support the microbial commonalities observed across diseases.

Considering the depletion of SCFA producers, several studies have performed metabolomics to directly assess the concentrations of major SCFA (acetate, butyrate, propionate) in IBD intestinal samples. In stool, there is a consistent depletion of butyrate in CD patients (134, 135) as detected via H-NMR. Interestingly, this observation is not translated to UC patients. Propionate and acetate are commonly found at lower abundances in IBD samples relative to controls, yet do not reach statistical significance (134, 136). Metabolism of these microbiota-derived metabolites can also be inferred via metagenomics of the microbes. Here, through sequencing of all microbial DNA in a stool sample, we can assess gene and pathway abundance as it pertains to a particular metabolic function or output. Through this approach, we can learn the potential function of a given microbiota. For example, Imhann et al. showed that multiple metabolic pathways are differentially abundant between IBD samples and control samples (137). Particularly, propionate metabolism KEGG pathways are decreased in both UC and CD samples, compared to controls. Butyrate metabolism and fatty acid metabolism KEGG pathways are decreased in CD patients, relative to controls. While certain microbial signatures have been associated with CD disease severity (138), this study did not find any significant KEGG pathways increased or decreased related to the clinical disease activity score (Harvey-Bradshaw Index, HBI) for CD (137).

## SUMMARY OF DISEASE-ASSOCIATED MICROBIOTA

While IBD and CF are two distinct diseases with unique genetic dispositions, diagnoses, prognoses, clinical manifestations, and treatment options, it is important to note that the gut microbiota contributes to clinical outcomes and quality of life in both cohorts. As discussed above and summarized in Table 1, there is an abundance of microbial signatures of dysbiosis that are shared between IBD and CF, suggesting the significant and important role that the gut microbiota plays in the trajectory of each disease. In fact, studies have shown that stool gut microbiomes from cwCF exhibit similarities to those in CD samples that can be directly compared using the CD Microbial-Dysbiosis Index (CDI) (30, 52). Interestingly, a high CDI, indicative of a gut microbiota very similar to that observed in CD, is positively correlated with levels of the inflammatory marker fecal calprotectin (52). Furthermore, longitudinal analyses of stool samples in cwCF across the first 4 years of life suggest that cwCF with a high CDI early in life (<2 years) have an altered microbiota later in development, marked by decreases in *Bacteroides* and increases in *Bifidobacterium*, which is negatively correlated with age (30). These data quantify the similarities between CD and CF on the microbiota level and highlight correlations with inflammation and altered microbiota development.

**TABLE 1:** Summary of intestinal microbiota in CF and IBD

Disease	Domain	Phylum	Family	Genus	Species	Lit. cited
CF	Bacteria	­↑Proteobacteria	↑­Enterobacteriaceae	­↑*Escherichia-Shigella*	­ ↑*E. coli*	(24, 25, 29, 31, 34, 35, 38, 41, 44)
			↑­Pseudomonadaceae	↑­*Pseudomonas*		(40, 52)
		↓Bacteroidetes	↓Bacteroidaceae	↓*Bacteroides*	↓*B. ovatus* ↓*B. vulgatus* ↓*B. xylanisolvens* ↓*B. intestinalis* ↓*B. caccae*	(34)
			↓Prevotellaceae	↓*Prevotella*	↓*P. copri*	(36, 39)
			↓Odoribacteraceae	↓*Odoribacter*		(36, 38)
		Firmicutes	↓Ruminococcaceae	↓*Faecalibacterium*	↓*F. prausnitzii*	(29, 31, 34, 37, 39)
				↓*Ruminococcus*	↓*R. bromii* ↓*R. albus* ↓*R. lactaris* ↓*R. obeum* ­↑*R. gnavus*	(31, 34, 39)
			↓Lachnospiraceae	↓*Roseburia*		(25, 36, 38)
				­↑*Blautia*		(31, 38)
			↓Eubacteriaceae	↓*Eubacterium*		(35, 37)
			↑­Enterococcaceae	­↑*Enterococcus*		(31, 35, 36, 38)
			­↑Streptococcaceae	­↑*Streptococcus*		(32, 34, 44)
			­↑Staphylococcaceae	­↑*Staphylococcus*		(37)
			­↑Veillonellaceae	­↑*Veillonella*		(25, 35, 38, 44)
				↓*Clostridium* cluster XIVa		(45)
		Actinobacteria	­↑Bifidobacteriaceae	­↑*Bifidobacterium*	­↑*B. longum* ­↑*B. breve*	(24, 29)
			↓Eggerthellaceae	↓*Eggerthella*		(34, 37, 44)
		↓Verrucomicrobiota	↓Akkermansiaceae	↓*Akkermansia*	↓*A. municiphila*	(34, 35, 44)
CF	Archaea		↓Methanobacteriaceae	↓*Methanobrevibacter*		(48)
CF	Eukarya	­↑Ascomycota		­↑*Saccharomyces* ­↑*Candida*		(29)
IBD: CD and UC	Bacteria	Proteobacteria	­↑Enterobacteriaceae	­↑*Escherichia* ­↑*Salmonella* ­↑*Klebsiella*	­↑*E. coli* ­↑*E. coli pks*+ ­↑AIEC	(60, 61, 77–80, 82, 84–88, 96–98)
			­↑Pseudomonadaceae	­↑*Pseudomonas*		(90)
			­↑Campylobacteraceae	­↑*Campylobacter*		(91–93)
			­↑Desulfuromonadaceae	­↑*Desulfuromonas*		(62, 101)
			­↑Desulfovibrionaceae	­↑*Desulfovibrio* ­↑*Bilophila*		(62, 101)
		Bacteroidetes	­↑Bacteroidaceae	­↑*Bacteroides*	­↑Enterotoxigenic *B. fragilis*	(74–76)
		Firmicutes	↓Ruminococcaceae	↓*Faecalibacterium*	↓*F. prausnitzii*	(62, 64–69)
			↓Lachnospiraceae	*↓Dorea* ↓*Anaerostipes*		(68)
			↓Oscillospiraceae	↓*Butyricicoccus*		(68)
			­↑Enterococcaceae	­↑*Enterococcus*	­↑*E. faecalis*	(65, 78)
			Clostridiaceae	­↑*Clostridium* ↓*Clostridium* cluster XIVa	­↑*C. difficile* ↓*C. coccoides* group	↑ (82, 99, 100) ↓ (66, 114)
			­↑Coriobacteriaceae	­↑*Atopobium*		(103, 104)
		Actinobacteria	↓Bifidobacteriaceae	↓*Bifidobacterium*		(66, 73)
			↓Coriobacteriaceae			(68)
			↑­Mycobacteriaceae	­↑*Mycobacterium*	­↑*Mycobacterium avium paratuberculosis* (*MAP*)	(94, 95)
		Verrucomicrobiota	↓Akkermansiaceae	↓*Akkermansia*	↓*A. muciniphila*	(63, 70–72)
IBD: UC and long-term CD	Archaea	↓Methanobacteroita ­↑Methanobacteroita	↓Methanobacteriaceae ↑Halobacteria (class)2^2^ ↑Thermococcales (order)2^2^	↓*Methanobrevibacter*	↓*Mb. Smithii^1^*	^1^ (123, 124) ^2^ (120–122)
		­↑Thermoproteota				(120–122)
IBD: UC and CD	Eukarya	↓Ascomycota		↓*Saccharomyces1^1^* ↓*Malassezia1^1^* ­↑*Candida2^2^*	­↑*Candida albicans^2^*	^1,2^ (127) ^2^ (131)
		­Basidiomycota				(127)
CD	Bacteria	Bacteroidetes	Bacteroidaceae	↑­*Bacteroides* ↓*Bacteroides*	­↑*B. fragilis^1^* ­↑*B. vulgatus^1^* ↓*B. fragilis^2^* ↓*B. vulgatus^3^*	^1^ (107) ^2^ (109–111) ^3^ (112, 113)
		Firmicutes	↓Veillonellaceae	↓*Dialister*	↓*Dialister invisus*	(66)
			↓Ruminococcaceae	↓*Ruminococcus*		(65, 106)
			↓Eubacteriaceae			(65)
UC	Bacteria	Bacteroidetes	­↑Bacteroidaceae1^1^ ↓Bacteroidaceae2^2^		↓*B. vulgatus^3^*	^1^ (107, 114, 116, 117) ^2^ (118) ^2,3^ (119)
		Actinobacteria	↓Bifidobacteriaceae			(115)

Our analyses pose the following questions. Why do two seemingly distinct pathologies, with independent genetic predispositions, adopt similar gut microbiota? Can we study these gut microbes to infer changes in host physiology? Next, we explore key physiological features of the intestinal milieu in both CF and IBD.

## INTESTINAL PHYSIOLOGY ACROSS DISEASE: CF AND IBD

### Inflammation

It is nearly universal for pwCF to have intestinal inflammation (6, 139, 140). An early study used whole gut lavage to quantify inflammatory markers in healthy controls and in cwCF, who either were clinically well or had fibrosing colonopathy (i.e., inflammation and scarring of the colon) (141). Here, the study reported a significant increase in intestinal albumin, IgG, IgM, eosinophil cationic protein (ECP), neutrophil elastase, IL-1β, and IL-8 in all CF samples, regardless of clinical presentation (141). Using capsule endoscopy, another group confirmed various pathologies in the jejunum and ileum of pwCF aged 10–36 years. These pathologies include villous blunting, edema, erythema, denuded mucosa, and mucosal breaks, in the form of erosions or ulcers (139). These markers did not correlate with a specific CF genotype; however, 70% of the sampled pwCF were pancreatic insufficient (PI). Similarly, fecal calprotectin levels were significantly higher in the PI CF cohort, compared to the pancreatic-sufficient CF cohort and the healthy controls (139). Calprotectin is secreted by active neutrophils and is a widely used marker of high mucosal inflammation (142), and numerous studies have used fecal calprotectin as a marker of inflammation in CF cohorts (34, 35, 41, 140, 143–149). Consistently, fecal calprotectin levels are elevated in CF populations and correlate with other variables, including quality of life scores (145, 146), CF genotype (140), fecal fat content (34, 41), antibiotic use (150), proton pump inhibitor use (151), weight and height (149), microbial markers of dysbiosis, notably increased pathogens (34, 35, 41) and microbial predicted KEGG pathways, including carbon fixation, phenylalanine metabolism (35), and fatty acid catabolism (34). Other markers of inflammation demonstrated to be higher in CF intestinal samples include S100A12 (calcium-binding calgranulin C, expressed in phagocytes, associated with inflammation and a proposed CF systemic inflammatory biomarker) (152, 153), lactoferrin, IL-1, tumor necrosis factor-aα (TNF-aα), rectal nitric oxide (NO) (43, 152), lipocalin-2, zinc-alpha2 glycoprotein, orosomucoid1 (47), and M2-pyruvate kinase (M2-PK) (35, 154, 155).

IBD is characterized as chronic inflammation in the GI tract (156), and there has been extensive research aimed at identifying approaches to quantify and describe the severity of inflammation. Aside from performing endoscopies to visualize the pathology of the intestinal lining, an invasive and uncomfortable procedure, key biomarkers of inflammation can be quantified in other sample types obtained from patients with IBD. Interestingly, one study showed that incidence of several matrix metalloproteinases (MMP-9, MMP-2, and MMP-9 complexed with neutrophil gelatinase-associated lipocalin [NGAL]) is increased in urinary samples from children with IBD and could be used as predictors for disease (157). MMPs are a family of metal-dependent enzymes that degrade and remodel the extracellular matrix of cells and may be implicated in IBD through mediating mucosal breakdown in response to enhanced inflammatory cascades (157). These proteins are already implicated in the development of cancer (157–160). Finally, like CF, fecal biomarkers of inflammation are also increased in IBD. Shared fecal biomarkers include calprotectin (156, 161–163), S100A12 (164), lactoferrin (156), NO production (165–169), and lipocalin-2 (156, 170, 171). Furthermore, serological markers of systemic inflammation are elevated in IBD, including C-reactive protein (CRP) (172, 173), TNF-aα (174), IL-10 (175), and suppression of tumorigenicity 2 or ST2, an IL-1 receptor (176).

One study directly compared fecal calprotectin levels from a cohort of children with CF, CD, and healthy controls (149). Here, they found that cwCF have lower levels of fecal calprotectin (94.3 mg/kg) than children with CD (2,133 mg/kg), but significantly more than healthy children (26.7 mg/kg). As observed previously (139), fecal calprotectin levels are largely dependent on pancreatic status. This study also cross-examined S100A12 and osteoprotegerin levels in CF, CD, and healthy stool samples (149). Both markers were significantly elevated in CD stool compared to both CF and healthy, suggesting that pwCD have more inflammation than pwCF, as measured through numerous markers. These differences may be study-dependent, but also reflect the lack of overt GI symptoms as reported by the CF group. For example, the majority of cwCF did not report the typical symptoms associated with intestinal inflammation, such as those seen in IBD (e.g., diarrhea with blood and mucus) (149) and adults with CF (177). Thus, a larger comparative study is needed to directly compare across diseases. 

### pH

The pH balance of the proximal intestine is largely regulated by the secretion of hydrochloric acid (HCl) and bicarbonate (HCO_3_^-^) (178). One of the major transporters of chloride and bicarbonate is the cystic fibrosis transmembrane conductance regulator (CFTR) (179). In healthy individuals, *cftr* is expressed at low levels in the stomach, but more strongly expressed along the intestinal tract (180). At the transcript level, *cftr* is highest in the duodenum with decreasing expression until the ileum. In the large intestine, *cftr* is expressed at moderate levels (180). Moreover, *cftr* expression was detected at higher levels in the crypts than villi (181). More recent data suggest that mRNA levels may not fully represent the amount of protein being translated, as CFTR protein levels were higher than predicted in villi when measured via immunolocalization (182). When functional, CFTR helps to neutralize stomach acid by secretion of bicarbonate to support optimal activity of digestive enzymes and bile salts (183, 184). In CF, where this protein is dysfunctional or absent, gastric acid is not properly neutralized, which leads to poor digestive function and nutrition, largely through the delayed or aberrant digestion of fatty acids in the proximal small intestine (183). Measurement of pH in pwCF through endoscopy has shown that the proximal small intestine is more acidic for a longer time in this cohort (185–188). For example, the mean pH in the distal duodenum in healthy individuals ranges between 6 and 6.5, while the range in CF is 4.5 and 5, depending on fat malabsorption status (186). In the colon, pH can be modulated through additional factors, including lactate production and SCFA metabolism, both of which are correlated with lower pH (39). As discussed previously, SCFA are decreased in CF relative to healthy controls, and lactate may accumulate as a result of pancreatic insufficiency and altered microbiota (189); thus, it would be hypothesized that altered bicarbonate flux coupled with altered metabolite concentrations would lead to lower pH in the CF colon. Indeed, colonic pH measurements in CF subjects are consistently lower (median ~6.5) than those in healthy controls (median ~7.5), along with levels of bicarbonate in the distal colon (190).

In IBD, the pH of the GI tract is similarly decreased relative to healthy controls (191–194). Across multiple studies and summarized in Nugent et al., the mean pH of the right (ascending) colon in healthy cohorts is ~6.2, while the right colon in UC cohorts is ~5.5 (191). Bicarbonate concentrations and secretion are both decreased in patients with active UC, which could account for the acidic colonic environment (191, 192, 195). This relative decrease in pH extends to the transverse and descending colon in IBD patients (196). More recent findings implicate a bicarbonate transporter, SLC26A3, in the electrolyte transport abnormalities and subsequent alteration in luminal pH in IBD (197). Specifically, *slc26a3* has reduced gene expression in the inflamed colonic epithelium, resulting in reduced electrolyte and fluid absorption. In a knockout mouse model of this transporter, there is increased permeability via decreased expression of tight junction proteins, alterations to the gut microbiota characteristic of IBD, and moderate increases in pro-inflammatory cytokine expression (198). These data suggest another mechanism by which inflammation may drive changes in luminal alkalinization and other pathologies associated with IBD. Overall, while the mechanisms appear distinct, the gut in CF and IBD is characterized by a more acidic pH.

### Mucus

CF is a disease characterized by thick, sticky mucus accumulation on various soft tissues. Loss of CFTR function results in a dehydrated luminal environment as bicarbonate facilitates adequate fluid volume and flow (179). Bicarbonate has also been shown to be crucial to the normal expansion and solubility of intestinal mucus (183, 199, 200). The most serious and prevalent acute complication of the intestine in CF is obstruction of the terminal ileum or proximal large intestine, which occurred in ~12% of infants with CF born in 2023 (177, 183). This form of obstruction is known as meconium ileus. Intestinal obstruction is not limited to the young ages; just over 1% of all adults with CF experience distal intestinal obstruction syndrome (DIOS) (177). The putty-like material that forms the obstruction in DIOS is a mixture of mucus and fecal-type matter with high microbial load (183, 201). Both meconium ileus and DIOS can be treated with osmotic agents (202); however, in severe cases, surgery is required.

The main components of the mucus layers are composed of mucins: high-molecular weight, heavily glycosylated O-linked glycoproteins, which are regulated through the control of 20+ mucin-encoding genes (203). MUC2 is the main mucin secreted in the small intestine and colon (204, 205), although other MUC genes contribute to mucin production (MUC1, MUC3, MUC4, MUC5B, MUC6, MUC11, and MUC12) (206). Dehydration of the mucosal surfaces, as seen in CF, alters mucin properties to a denser, more highly glycosylated (with fucose, galactose, and N-acetylglucosamine) and sulfated form, compared with healthy controls (203, 207, 208). In these studies, there was no reported difference in the amino acid composition of control and CF small intestinal mucins (207, 208). Bicarbonate availability plays a role in mucin’s biochemical and biophysical properties (209). Under normal conditions, bicarbonate ions in the intestinal milieu sequester cations (e.g., Ca^2+^) from binding previously packed mucins in the secretory granule. Upon this sequestration of cations, the pH is increased, which triggers the rapid expansion and hydration of the mucin polymers (200, 204, 209–211). In bicarbonate-limited environments, such as in CF, cations remain bound to packed mucins, and these mucin complexes are not completely expanded (199), resulting in highly viscous mucus, which likely is what leads to intestinal blockage. In CF mouse models, this compacted mucus layer in the ileum is two to three times denser than that measured in WT mice (209). Interestingly, the expansion of the mucin can be rescued with the addition of bicarbonate (209). These findings highlight the complex interplay that CFTR function has on mucin formation and function.

The intestinal mucosal layer in IBD is compromised, relative to healthy controls. Particularly in UC patients, the mucosal layer in the colon is thinner, with a reduction up to 70% (212) compared to healthy and CD layers (213). This compromised mucosal layer is linked to the depletion of goblet cells in the UC colorectal mucosa, which are responsible for the production and secretion of mucin (214), reduced expression of MUC2 (215), defective polymerization of secreted mucin (212), and a fourfold increase in proteolytic activity in the UC colonic lumen (212). The composition of the mucins in UC is altered to adopt an increase in sialomucins and reduction in sulfomucins, both of which are linked to the degree of inflammation (206, 216–218) and are reversible upon remission (219).

In CD patients, the pathology of the intestinal tissue is apparent. Inflammatory ulceration of the colon mucosa with moderate epithelial damage and structural changes is frequent (215). Goblet cell count and mucus production are largely unchanged (215). There is great heterogeneity in transcription of various MUC genes, likely due to sampling region and the widespread nature of the disease; however, overall, there is a reduction in expression of MUC1, MUC3, MUC4, and MUC5B in CD (215, 220). Interestingly, MUC2, the main mucin secreted, is not differentially expressed. It is hypothesized that the alternative MUC genes may possess additional functions to secreting mucin, since non-mucus-secreting cells express these genes (221, 222). Furthermore, there is a reported imbalance in glycosylation with the prevalence of weakly sulfated mucins in CD patients (215, 223).

Taken together, these findings suggest key differences in mucosal layers between CF and IBD, and within types of IBD. First, CF is characterized as having a thick and dense mucosal layer, comprised of mucins that are not expanded nor hydrated, with highly glycosylated and sulfated modifications. Collectively, this contributes to a static mucosal layer that hinders motility and transit time and provides sustained nutrition to the gut microbiota. Conversely, IBD is characterized as having a disrupted mucosal layer, with decreased secretion of mucins in UC, coupled with differential glycosylation and sulfation patterns in both UC and CD. With less mucin, the colonocytes are exposed to the gut microbiota and their products, inducing inflammatory responses. Altered glycoproteins contribute to the variability of the protective function of mucins. These features of mucin and the mucus layer appear to be a key difference between CF and IBD.

### Bile

Bile acids, produced and secreted by the liver, are essential for the emulsification of dietary fat, cholesterol, and lipid-soluble vitamins (224), in addition to acting as bioactive signaling metabolites in the regulation of their metabolism (225), glucose metabolism (226), liver regeneration (227), and innate immunity (228, 229). As CFTR is expressed in the biliary tree, hepatobiliary function is disrupted in CF (230–234). The most common manifestations of CF-related liver disease (CFLD) include focal biliary cirrhosis, liver steatosis, secondary sclerosing cholangitis, and liver cirrhosis leading to hypertension (235). Additional indicators of disease include altered bile acid metabolism and an impaired hepatobiliary circuit, both of which result in nutrient malabsorption and excessive fecal bile acid excretion (224, 230, 236, 237). In humans and CF mouse models, this excretion surplus can be up to threefold higher than observed in non-CF humans and mouse models (231, 238–240). There could be numerous explanations for the increased excretion of bile acids in CF stool: (i) altered farnesoid X receptor (FXR) signaling, a negative feedback cascade that ultimately regulates bile acid synthesis (240), (ii) hindered absorption of bile acids in the ileum and colon due to the thickened mucosal layer (241), (iii) altered functionality of the apical sodium-dependent bile acid transporter (ASBT), an active transporter of bile acids largely active in the ileum (242), and (iv) the prevalence for small intestine bacterial overgrowth (SIBO) in the CF population (183, 243).

In addition to increased excretion of total bile acids, the composition of bile acids in the CF intestine is shifted compared to healthy controls (224, 238, 244). In general, pwCF have a higher concentration of primary bile acids, largely driven by cholic acid (224, 238, 244, 245). These forms of bile acids are thought to be more toxic and inflammatory at high levels due to the detergent-like activity on membranes (246). In cwCF, there is a similarly observed increase in primary bile acids, specifically primary unconjugated bile acids (G. A. O’Toole et al., unpublished data). There is also a concomitant decrease in selected secondary bile acid abundance (O’Toole et al., unpublished), which are metabolized from primary bile acids by the gut microbiota through various enzymatic reactions (247). In a cohort of 18 non-CF and 18 CF stool samples, lithocholic acid (LCA) and its secondary metabolites (e.g., alloisolithocholic acid, taurolithocholic acid) are significantly depleted in CF stool (O’Toole et al., unpublished). These secondary bile acids and secondary metabolites are derived from the primary bile acid chenodeoxycholic acid (CDCA) and have anti-inflammatory properties (248, 249). These trends of increased primary bile acids in stool track with serum measurements, as well (224), where pwCF with severe disease have increased concentrations of cholic acid in serum. These data suggest the use of serum bile acids as a biomarker for severe disease. Interestingly, conjugation status of bile acids is also altered in CF, where there is an increase in glycine-conjugated bile salts, rather than taurine-conjugated (224, 245). This may indicate a change in amino acid availability in the CF gut and/or altered deconjugation activity by gut microbiota. Taurine has associated anti-inflammatory, anti-apoptotic properties, and its supplementation may aid in fat absorption in CF (250–252) and decrease overall inflammation in colitis models (253, 254). A decrease in taurine-conjugated bile salts may simply compound existing intestinal inflammation in CF.

These signatures of bile acid dysbiosis are also observed in IBD. While total fecal bile acid concentrations are similar to those measured in healthy controls (248), the composition of bile acid pools shows increased primary bile acid species (136, 248), conjugated primary bile salts (136), and sulfated bile acids, especially for those with active disease (248), and decreased secondary bile acids, largely driven by reduced lithocholic acid and deoxycholic acid (136, 248). The reduction in secondary bile acids in fecal samples aligns with what is seen in serum (248). Because CD and UC are associated with symptomatic diarrhea, a possible explanation for a reduction in secondary bile acids may be that the transit time through the colon is too short for these compounds to be metabolized by the microbiota (248, 255). However, another study examining the abundance of microbial genes related to bile acid metabolism reported that IBD microbiomes, specifically CD microbiomes, have decreased potential for deconjugation of primary bile acids via bile salt hydrolase (BSH), the first step in bile acid metabolism (256). Furthermore, they report that the decrease in BSH relative abundance is attributed to taxa in the phylum Firmicutes (256). These findings suggest the direct role of the microbiome in bile acid dysmetabolism in IBD. Overall, the shift in bile acid species in IBD parallels that of CF.

### Fats

Pancreatic insufficiency is observed in nearly 85% of pwCF, which results in inadequate production and secretion of essential digestive enzymes, aberrant neutralization of gastric acid, and altered hydration (183, 257). Coupled with altered bile acid profiles, the breakdown and absorption of fats and other lipid-soluble compounds are largely impaired in CF (183, 258, 259). The accumulation of undigested lipids in the intestine contributes to increased risk of steatorrhea (260–262), pulmonary symptoms (263), and microbial dysbiosis (264, 265). Over the years, many groups have investigated the lipid profile in CF serum to address the severity of fat malabsorption (259, 266–271). There has been a consistent depletion of linoleic acid (LA) and docosahexaenoic acid (DHA) observed in serum samples isolated from pwCF (272–274). LA and DHA are key components of membrane phospholipids, in which the composition contributes greatly to proper membrane function, transport, and metabolism in all cell types. Thus, the depletion of these fatty acids in CF serum may indicate one factor explaining the disturbed channel localization and activity (259). The essential fatty acid (EFA) deficiency varies depending on genotype and diet (275, 276). For example, CFTR heterozygotes possess altered fatty acid profiles compared to controls, suggestive of a mild fatty acid metabolism defect associated with the disease (277). A decrease in linoleic acid is usually inversely correlated to arachidonic acid (AA) concentrations (263). AA is a precursor to pro-inflammatory molecules like prostaglandins and leukotrienes and a stimulator of mucus production (278); an imbalance in AA metabolism may sustain or compound existing CF inflammation and pathologies (279, 280). In addition to fatty acids, ceramide profiles are altered in CF serum samples, whereby there is an increase in very long-chain and long-chain ceramides (281). This observation, coupled with decreased plasma cholesterol levels (282), may influence the stability of rafts in cell membranes and inflammation (259).

Only recently has the lipidome in CF stool been reported (265). Here, the authors aimed to draw associations between the fecal lipid profile and changes in the microbiota, a link that has been explored in intestinal carcinogenesis (264). Overall, children with CF have significantly increased levels of triglycerides, diacylglycerols, monoacylglycerols, and fatty acids in their stool (265). Asensio-Grau et al. note that as total fat species increased, there was a positive correlation with levels of Proteobacteria (*Escherichia-Shigella*) and a negative correlation with Bacteroidetes (*Bacteroides*) and Verrucomicrobiota (*Akkermansia*) (265). Notably, the CF lipidome correlated with pH, whereby the samples with the highest concentrations of fat had the lowest pH. CF stool with the most abundant lipid profiles also negatively correlated with height and weight percentiles (265). These results support the understanding that altered fat digestion and absorption, as indicated by high triglyceride and high fatty acid concentrations, respectively, contribute to altered microbial composition in the intestine, abnormal intestinal physiology (e.g., pH, fecal fat content), and nutritional status indicators (e.g., weight, height).

In IBD, fat profiles in both serum and colonic mucosa are altered (283). In serum isolated from IBD patients, there is a significant depletion of certain polyunsaturated fatty acids, including alpha-linolenic acid, eicosadienoic acid (284), and linoleic acid (285, 286). Inversely, there is an enrichment of saturated fatty acids, particularly driven by 16-carbon chain palmitic acid. In colonic tissues, there is a similar enrichment of saturated fatty acids, with additional contribution from 12-carbon (lauric acid) and 18-carbon (stearic acid) fatty acids. There is also an observed depletion in monounsaturated fatty acids (C18:1: oleic acid), polyunsaturated fatty acids (C18:2, C18:3, and C20:5: eicosadienoic acid) in colonic tissues isolated from IBD patients. The decrease in polyunsaturated fatty acids in IBD negatively correlates with histological measures of inflammation assessed by the Geboes activity index, whereas the enrichment of saturated fatty acids positively correlates with the Geboes activity index (284). Similar to what is observed in CF samples, arachidonic acid (AA) is enriched in both CD and UC samples (136, 285, 287). This enrichment is associated with colorectal cancer progression (288). Furthermore, glycerol concentrations are higher in stool from CD patients, compared to healthy controls and UC patients (134). Interestingly, many acylcarnitines, which essentially transport fatty acids to the mitochondria, are enriched in CD (136), indicative of the host’s use of enriched fatty acids for energy under inflamed states. Thus, some shared features of altered fat metabolism seem to be common to CF and IBD.

### Oxygen and oxidative stress

As discussed above and summarized in Table 1, both the CF and IBD microbiotas adopt a bacterial community that favors facultative anaerobes, such as those in the Enterobacteriaceae family, and excludes a number of strict anaerobes, such as SCFA-producer *Faecalibacterium prausnitzii*. This consistent observation is conserved across various inflamed-gut contexts (e.g., obesity, antibiotic exposure, colorectal cancer, celiac disease) (289–291) and has sparked much speculation as to oxygen gradients in the intestine in the context of inflammation (292, 293). Under healthy conditions, aerotolerant bacteria consume oxygen in the intestinal lumen, rendering this niche essentially anoxic. A steep oxygen gradient exists between the lumen and epithelial tissue, whereby the oxygen-containing layer adjacent to the colonic tissue is very thin (294). However, under inflamed states where an increase in aerotolerant microbes is detected, there is reason to explore the role of increased oxygen in pathology. The oxygen hypothesis posits that chronic inflammation of the intestine induces release of oxygen in the form of oxygen-bound hemoglobin from blood, increasing overall pO_2_ in the intestinal lumen (292). Other modes of oxygen introduction under inflammation include epithelial hyperplasia that increases the number of immature normoxic colonocytes and neutrophil transmigration across the epithelia and subsequent activation, releasing reactive oxygen species (ROS), usually in the form of hydrogen peroxide, into the intestinal lumen (295–298). This proposed shift in oxygen tension, coupled with increased production of ROS and reactive nitrogen species (RNS) and the byproducts generated by their degradation (i.e., nitrates, nitrites), can drive changes in gut microbiota structure in certain microenvironments observed across numerous intestinal inflammatory diseases and models (295–297, 299, 300). Furthermore, methanogens and acetogens (archaea and bacteria, respectively) highly sensitive to trace amounts of oxygen, as well as obligately anaerobic sulfate-reducing bacteria, are significantly depleted in the homozygous CF colon compared to healthy controls (Table 1) (48). Together, these data support the idea that oxygen may be a contributing factor for microbial dysbiosis in the CF gut; however, the role of oxygen has yet to be directly investigated in the context of CF.

While oxygen concentrations and pO_2_ have not yet been directly measured in the CF gut to our knowledge, factors involved in the detoxification of ROS have been investigated in the context of CF. First, CFTR is implicated in the regulation and transport of more than just bicarbonate and chloride; it is also involved in the transport of nitrate, formate, acetate, and glutathione (GSH) (301–303). Glutathione/glutathione disulfide (GSH/GSSG) couple is one of the most important pools of cellular redox systems and is the first line of defense against free radicals (304, 305). Studies report low levels of GSH in the plasma and neutrophils of pwCF (301). Second, maldigestion and intestinal malabsorption can lead to a reduction in lipid-soluble antioxidants (e.g., vitamin E, carotenoids, coenzyme Q10, and fatty acids) (302, 306, 307). With less abundant and functional detoxification mechanisms in CF, ROS and other forms of oxidative stress accumulate, leading to increased vulnerability to impaired lipid membranes, cell damage, and overall inflammation in CF cohorts.

The oxygen hypothesis is more nuanced in cases of IBD, where microbiological evidence suggests the role of increased oxygen and ROS; however, direct measurements of luminal oxygen suggest otherwise. As similarly seen in CF gut microbiota, Enterobacteriaceae and other facultative anaerobes are increased in IBD (see Table 1). The dysbiosis observed in CF and IBD shares similarities to those seen following hyperbaric oxygen therapy (HBOT) (294, 308). In addition, mouse studies have shown that *E. coli* utilizes a cytochrome oxidase, AppBCX, to colonize in the presence of colitis, suggesting the involvement of aerobic respiration in the microbe’s growth enhancement in the inflamed gut (299). Furthermore, the source of oxygen in this context is determined to be the host’s epithelial NADPH oxidase NOX1-derived hydrogen peroxide, which is subsequently detoxified using microbial catalase to generate oxygen and water. In support of these findings, another group has investigated the role of aerobic respiration in *Citrobacter rodentium* colonization in the inflamed mouse gut (300). This model is frequently used to investigate host-pathogen immune interactions in the gut and to understand the pathogenesis of CD and UC (309). Miller et al. report the use of *C. rodentium*’s cytochrome *bd* oxidase CydAB and cytochrome *c* peroxidase Ccp in its growth advantage in the inflamed gut (300). Again, the oxygen source is derived from host NADPH oxidase activity; the phenotype can be ablated using the NADPH oxidase inhibitor apocynin (300). Considering these findings, it is no surprise that supplementation of the probiotic *Lactobacillus*, which is catalase- and dismutase-positive, is effective in suppressing the inflammatory process in mouse models of IBD (310). These studies highlight the role of increased oxygen in the dysbiosis observed under inflamed states.

Inversely, there is evidence supporting the role of hypoxia, or lower oxygen tensions, under inflammation. Sites of inflammation have been associated with altered tissue metabolism, imbalances in tissue oxygen supply and demand, and the production of high concentrations of RNS and ROS (311, 312). Many of the shifts in energy expenditure are attributed to the recruitment of inflammatory cells, including neutrophils. Once at the site of inflammation, these immune cells have heightened demand for oxygen and other nutrients to facilitate proper phagocytosis and microbial killing (311), thus depleting local oxygen concentrations. These regions of inflammation-driven hypoxia have been measured *in vivo* in numerous studies (313–316). In addition, these studies and others have reported an increase in hypoxia-inducible factor (HIF) expression in sites of inflammation, specifically in the context of IBD (317–320); this response serves as an alarm signal for the resolution of inflammation and redirection of metabolism to better adapt to hypoxia (311). When HIF-1 is active in the intestine, it regulates many processes involved in barrier integrity, some of which include increased mucin production, xenobiotic clearance, and nucleotide metabolism and signaling (311). Functional HIF-1aα is protective against advanced colitis in mouse models (315), suggesting the critical role of hypoxia adaptation in intestinal inflammation.

Taken together, these findings suggest the complicated role that oxygen plays in intestinal inflammation. Microbiological analyses suggest the essentiality of adaptation to ample oxygen; however, direct measurements of oxygen levels propose hypoxic zones. Interestingly, HIF-1aα expression is markedly reduced in CF airway epithelia, a phenomenon that may apply to CF intestinal cells (321). These results would suggest an opposite response in CF compared to IBD. These conflicting observations reinforce a gap in understanding and underscore the need for further studies.

### Alternative nutrients

If oxygen is not the main driver of a more aerotolerant microbial community, then what is? As mentioned above, immune cells release large quantities of ROS and RNS and byproducts of their degradation, all of which can be stripped of their electrons and used for energy by microbes. A major byproduct of the inflammatory response is nitrate. Several studies have shown that nitrate levels rise rapidly in the inflamed colon following a reaction between a superoxide radical and nitric oxide to form peroxynitrite, which can be rapidly converted to nitrate (322). *E. coli* can utilize this nitrate as an alternative electron acceptor via nitrate respiration (323, 324), conserving almost as much energy as when oxygen serves as the terminal electron acceptor, whereby these genes encoding nitrate reductases are absent in many obligate anaerobes like *Bacteroides* and *Clostridium*. Through oxidative burst and subsequent oxidation, neutrophils also release thiosulfate and tetrathionate, both of which can be used as electron acceptors by *Salmonella* in the inflamed gut (105, 325). ROS and RNS have the capability of oxidizing organic sulfides (e.g., methionine) and tertiary amines (e.g., trimethylamine) to form S-oxides and N-oxides, respectively; these compounds can also be used by *E. coli* to promote its growth under inflammation (105, 322). Another metabolite that is increased in the context of inflammation is formate, which is largely microbial-derived (297). Data suggest that formate oxidation coupled with aerobic respiration by *E. coli* is an important pathway involved in the observed outgrowth of this microbe in the inflamed GI tract (297).

Fecal metabolite profiles observed in CF have further alterations than the ones noted above. Metabolomics has pointed to increases in gamma aminobutyric acid (GABA), choline, ethanol, propylbutyrate, and pyridine, with decreases in aromatic amino acid derivatives (e.g., sarcosine, 4-methylphenol, phenol, benzaldehyde), nucleotides (e.g., uracil), glucose, acetate, and methylacetate (37, 326). In addition to depletion in SCFAs in CF metabolomes, proteomics analysis demonstrates a reduction in pantetheine, which is required for the conversion of propanoate to propionyl-CoA (35, 47, 327). Because of increases in *C. difficile* abundances in CF microbiota, there is an inferred increase in this microbe’s biosynthesis of alcohols, esters, and pyridine (37). Furthermore, due to altered fat absorption, various vitamins are deficient in CF cohorts, including vitamins D (328), A, E, and K (329). Lastly, in one study, an increase in *Enterococcus* was associated with an enrichment in lactate in the CF microbiota (39). These observed shifts in metabolism likely contribute to alternative nutrient sources for resident microbes and the potential overgrowth of certain taxa.

Metabolic profiles are also altered in IBD. These profiles are characterized by increased levels of metabolites derived from the metabolism of aromatic amino acids, indicative of enhanced proteolytic fermentation metabolism (330, 331). Particular metabolites, such as p-cresol sulfate and 3-indoxyl sulfate, are increased in IBD gut metabolomes and are linked with colorectal cancer (332, 333). Amino acids, specifically taurine, and their derivatives are enriched in IBD metabolomes, supporting the proteolytic fermentation observation (331, 334). Conversely, feces from patients with IBD show decreases in vitamins B3, B5 (136, 335), and B7 (331), as well as nucleotides and enterolactone (331). Likely, the altered bacterial diversity associated with IBD contributes to the dysfunction of fiber digestion and vitamin production, as evidenced by functional outputs of IBD-associated microbial communities (336).

### Diet

A key contributing factor to altered stool lipidomes and metabolomes in CF populations is diet. Historically, a high-fat, high-energy diet has been implemented in young persons with CF to counteract the increased energy demands inherent to the disease (e.g., fat malabsorption, infection, and respiratory disease). Despite access to highly effective modulator therapy (HEMT), which may partially attenuate these energy demands, the traditional dietary regimen remains largely unchanged. Consequently, the CF population has a significantly higher energy intake (22). Specifically, cwCF consume higher quantities of fat (i.e., trans fat, saturated fat) and lower quantities of carbohydrates (i.e., starch, resistant starch, whole grains, fiber) and key vitamins and minerals (i.e., iron, magnesium, and vitamin B12) (22). Interestingly, one study has reported associations between dietary intake and certain bacterial genera in the intestine. Of note, fat intake is positively correlated with the abundance of *Subdoligranulum* (22), a recently discovered genus that produces SCFAs, is associated with *Akkermansia muciniphila,* and promotes a healthy metabolic status (337). While the metabolic capabilities of *Subdoligranulum* suggest its beneficial role in the intestine, fat intake has previously been associated with enrichments in *Escherichia-Shigella* and *Streptococcus* in the guts of cwCF (338). Such opposing findings underscore the need for further research to determine the interplay of fat metabolism, the gut microbiota, and host response in the context of CF.

In addition to CF, there is evidence in the IBD field that diet modulates gut inflammation and symptoms in this disease cohort, specifically in individuals with small bowel Crohn’s disease (339). While there is limited population-level data to elucidate a specific diet and its relation to CD risk (340, 341), individual dietary components have been probed in several studies. Of note, dietary fiber, specifically fruit fiber, has been associated with up to a 40% reduction in CD risk (342). Interestingly, this correlation did not extend to UC populations (342). Studies on dietary fat intake report mixed results, whereby some epidemiologic studies suggest associations between wide-scale intake of fat species (i.e., total fat, saturated fat, n-6 polyunsaturated fat) with increased CD incidence (343), yet other studies do not report any associations (344). Alternatively, intake of fat, including LCFAs (e.g., docosapentaenoic acid, eicosapentaenoic acid, and docosahexaenoic acid), has been associated with reduced risk of development of UC (344). In separate studies of adults with IBD, animal protein was associated with increased risk of IBD (345), whereby dairy consumption correlated with reduced risk of CD, specifically (346). Lastly, carbohydrate intake has not been associated with risk for IBD across numerous studies (347, 348).

Diet has been used as a therapeutic strategy in the management of IBD. Several diet regimens have been implemented to individuals with IBD—some with promising results. Exclusive enteral nutrition (EEN), a therapy of specialized liquid nutrition formulas and water for a 6- to 12-week duration, is an effective treatment for children with CD by means of inducing remission and promoting growth (349, 350). Due to the difficulties of tolerating and complying with a strict liquid diet, older children and adults with CD may prefer to adhere to partial enteral nutrition (PEN) regimens, which allow oral food intake with 50% of total calories supplemented with enteral formulas. While effectiveness is reduced compared to EEN (351, 352), PEN was better tolerated and resulted in a significantly higher rate of corticosteroid-free remission following therapy (339, 353). Other diets (e.g., CD-TREAT, Crohn’s Disease Elimination Diet [CDED], Specific Carbohydrate Diet [SCD], Fermentable Oligo-, Di-, Monosaccharides and Polyols [FODMAP]) focus on the exclusion of certain components that may be pro-inflammatory (i.e., gluten, lactose, alcohol) while maintaining macronutrients, vitamins, minerals, and fiber in quantities that are similar to those in EEN formulas. Early evidence suggests that exclusion diets, specifically CD-TREAT, induce changes to the fecal microbiome and metabolome similar to those observed following EEN (354). Similarly, these diets result in clinical response and higher remission rates in treated children with CD (355). Additional research is needed to address the efficacy and sustainability of such diet regimens across IBD cohorts. These results suggest the complex interplay between diet, nutrition, the gut microbiota, and metabolic output, all of which likely contribute to respiratory health and systemic inflammation in disease cohorts including CF and IBD.

### Antibiotics and other therapies

Individuals with each respective disease, CF or IBD, receive antibiotics in their typical treatment regimen. In CF, beginning at a young age, children acquire infections of the ear, sinus, and airway attributed to poor mucociliary clearance and thick mucosal secretions (356). Antibiotics are not only used to clear the infection but also improve lung function in this cohort (357). Individuals with CF receive antibiotics via oral ingestion, inhalation, and intravenously (IV), depending on the type and severity of infection (177). For context, 12.5% of CF patients were treated with IV antibiotics following a pulmonary exacerbation in 2023, a statistic that has been significantly declining since 2008 due to the widespread use of modulator therapy (177). More commonly, pwCF who are positive for a *Pseudomonas aeruginosa* lung infection are prescribed inhaled tobramycin (51.8% of patients) or inhaled aztreonam (31.9% of patients) (177).

For IBD, antibiotics are prescribed for treating luminal disease, fistulizing disease, bacterial overgrowth, or septic complications, such as abscesses and postoperative wound infections (358). In a study of nearly 3,000 IBD patients, overall antibiotic use was observed for 29% of those enrolled (30% in CD cohorts, 26% in UC cohorts) (156). In both disease cohorts, antibiotics are used as maintenance therapy (358–360). These staggering percentages simply underscore the ongoing need for antibiotics in IBD and CF, two diseases that pose an increased risk of infection.

While antibiotics are an essential component of the treatment regimen, numerous studies have shown that these compounds alter the gut microbiota (322, 358, 361–363) and render the individual at risk for heightened inflammation and recurrent infection (156, 358, 364). While alterations to gut microbiota observed post-antibiotic use depend on the type of antibiotic (365), there is typically a reduction in overall diversity, a decrease in Bacteroidetes and Firmicutes taxa (365), and an enrichment in Enterobacteriaceae and microbes harboring antibiotic-resistant genes (ARGs) (322, 365, 366). In addition, a study demonstrated that the cecal contents of mice treated with streptomycin increased in pH and oxidation-reduction potential (Eh), indicating that antibiotic exposure alters host physiology, likely via the gut microbiota (367). These immediate shifts in gut microbiome have long-lasting effects. In the context of CF, cwCF who took antibiotics during the first 6 months of life had altered gut microbiota at 2–4 years old (30). Taxonomical shifts include decreases in *Bifidobacterium, Blautia,* and *Streptococcus*, with increases in *Veillonella* and *Prevotella* (30). Because some of the long-term follow-up collections were outside of the canonical 3-year developmental window for the gut microbiome, it is hypothesized that these microbes may not recover to levels observed in the non-antibiotic cohorts. In the context of IBD, antibiotics are associated with GI disturbances and pose an increased risk for *C. difficile* infection (368), by which IBD patients suffer worse outcomes (369). Similar to what is observed in CF, there is an association with early antibiotic use and altered gut microbiota, increased mucosal microbial burdens, and increased CD occurrence (370), suggesting the long-term role that antibiotics have on gut microbiome development and maturation.

Another common treatment for persons with CF and IBD is corticosteroids. Corticosteroids can bind to the intracellular glucocorticoid receptor and enter the nucleus to produce anti-inflammatory effects (156, 371). pwCF receive inhaled corticosteroids more than oral corticosteroids, although the pulmonary guidelines recommend against their chronic use in the absence of asthma or allergic bronchopulmonary aspergillosis (ABPA) (177). Among the cwCF aged 0–3 years old, about 16% were prescribed inhaled corticosteroids (177). In general, there are inconsistent results pertaining to steroid therapy in the CF cohort: some studies show an increase in lung function following steroid use (372), while others found insufficient evidence to recommend corticosteroids to this population (373). What is consistently reported is the potential for adverse side effects (374, 375). In the IBD population, corticosteroids are widely used in patients with moderate to severe disease activity (376). It is important to note that IBD is characteristically diagnosed in adolescence and adulthood (55, 56), so the CF population is exposed to corticosteroid use earlier. Shortly after the diagnosis of IBD, patients are typically prescribed corticosteroids; one study of an inception cohort reported that 43% of CD patients and 34% of UC patients received steroids (377). The immediate outcomes are variable in the IBD population, whereby some individuals achieve complete remission (~60%), partial remission (~25%–30%), or have no response (10%–15%). After 1 year of corticosteroid use, about one-third of patients have prolonged response, while the remaining develop steroid dependence or require surgery (377). By contrast, while this treatment is not encouraged in the broader CF cohort, with exceptions for those with asthma or ABPA, cwCF are exposed to corticosteroids earlier in life than those with IBD, due to the differing times of diagnosis. Notably, a good proportion of the CF cohort receives corticosteroids during the critical development of their gut microbiomes.

Mouse models have shown significant effects of corticosteroids on the gut microbiota and intestinal gene expression (378). Specifically, wild-type (WT) mice treated with dexamethasone, a synthetic glucocorticoid that is routinely used to treat IBD (379), shift to a gut microbiome with lower Bacteroidetes abundances, increased Actinobacteria (*Bifidobacterium*) and Firmicutes (*Lactobacillus*), and a decrease in a mucin-degrading microbe found in the murine intestine, *Mucispirillum* (378). In parallel, steroid treatment in this mouse model significantly decreases gene expression of several mucin genes, such as *muc1*, *muc2,* and *muc3* in the proximal colon (378), suggestive of mucosal-associated microbiota alterations directly attributed to steroid treatment. Interestingly, children with severe UC who did not respond to steroid treatment exhibited a gut microbiota with reduced richness, or number of phylospecies (380), indicative of the necessity for gut microbes in the efficacy of steroids. While some associations suggest the interplay between steroid treatment and gut microbiota, there is limited research investigating the direct and sole impact(s) of corticosteroids on the gut microbiome, since patients in these cohorts are administered a slew of medications, which likely confound observed associations.

There are several treatments that are specific to CF cohorts, including mucus thinners, bronchodilators, and CFTR modulator therapy, which comprises two CFTR correctors and one CFTR modulator, either individually or in combination. Highly effector modulator therapy (HEMT) is the triple drug combination, elexacaftor-tezacaftor-ivacaftor, abbreviated ETI. Due to the increased accumulation of viscous mucus in organs due to CFTR dysfunction and subsequent risk of obstruction, many individuals with CF are prescribed mucolytic medications, such as Dornase alfa (DNase) and hypertonic saline, and bronchodilators, such as beta-agonists, to improve lung function and reduce the risk of infection (177). Often, pwCF are taking multiple of these therapies at the same time (177). While there is limited evidence of the effects of these medications on the gut microbiota specifically, it is inferred that mucolytics like Dornase alfa may break down extracellular DNA in the intestine and alter intestinal mucus secretion and/or composition, thereby improving motility. However, it is not known how much inhaled Dornase alfa reaches the intestine. Beta-agonists are largely used in CF to relax the smooth muscles lining the airway, allowing for bronchodilation and better lung function (381). *In vitro* studies with intestinal organoids reveal a positive correlation with beta-agonist treatment and CFTR function, even in organoids with gene mutations that render CFTR with minimal function (382). However, like mucolytics, there remains a gap in knowledge pertaining to the role of beta-agonists in gut microbiota structure and function.

In the era of CFTR modulators, there have been incredible reports of life-changing improvements in lung function, nutrition, inflammation, GI function, and quality of life (231, 383–391). In the context of the CF gut, HEMT has been associated with increased alpha diversity measures, increased abundance in SCFA-producing bacteria, and decreases in *Enterobacteriaceae* and oral microbes detected in stool (383, 388). Antibiotic use post-HEMT is also significantly reduced and associated with increases in SCFA-producer *Roseburia* (388). Subsequently, ARG abundance is reportedly decreased following HEMT; when these ARGs are subset by antibiotic class, the abundance of ARGs conferring resistance to peptide antibiotics is significantly decreased (388). Overall, fecal calprotectin levels are reduced in cwCF following HEMT (383, 388), and there is a modest reduction in CDI, the Crohn’s Disease Index of inflammation-associated microbial dysbiosis (388). Interestingly, metagenomic sequencing reveals alterations in microbial metabolic pathways, with an increase in amino acid biosynthesis and decreases in aerobic respiration, oxidative phosphorylation, and acid tolerance (388). These results suggest an altered intestinal environment and the greater capacity for microbes to synthesize a diversity of metabolites. While these shifts are promising, one study advised that significant differences in microbiota structure and function persist between non-CF and CF cohorts, even when those with CF were on extended HEMT therapy (387). Another study noted no significant changes to lipid profiles in stool from CF patients on CFTR modulators (265). Thus, ongoing research is required to clarify the long-term and longstanding effects of CFTR modulators and to properly identify outstanding concerns, as they relate to GI function.

Canonical treatments for IBD include 5-aminosalicylic acid (5-ASA), Janus kinase (JAK) inhibitors, anti-tumor necrosis factor (TNF)-aα, IL-12/23 antibodies, and anti-integrin antibodies, which all mostly target effector immune responses (392). Determination of which treatment to use is dependent on stage, extent, and severity of disease (393). These therapies can induce remission in patients with IBD, yet are associated with severe side effects and lower quality of life scores (392). In addition, there are variations in efficacy for many of these therapies with inconclusive mechanisms as to why (393). In the case of 5-ASA, which is an anti-inflammatory drug known to inhibit the production of cytokines via peroxisome proliferator-activated receptor-gamma (PPAR-gamma) (394), over half of IBD patients fail to respond to this treatment, or lose response over time (395, 396). This variability in clinical efficacy has been partially attributed to the gut microbiome. In one study of a UC cohort, the gut microbiota of adults treated with 5-ASA for at least 1 month shifted to adopt increased abundance of Firmicutes (i.e., *Faecalibacterium*) and reduced abundance of Proteobacteria (i.e., *Escherichia-Shigella*) (397). In this regard, several studies have shown that stool cultures can metabolize 5-ASA into *N-*acetyl-5-ASA, a form of the compound that lacks anti-inflammatory activity (398, 399). It has been identified that members of the phyla Proteobacteria and Firmicutes possess this enzymatic activity (400–402). Recently, specific microbial enzymes were identified to be necessary for this metabolic conversion and subsequent inactivation of 5-ASA (402). Prevalence and abundance of these enzymes, as measured through stool metagenome sequencing, are associated with a greater risk of treatment failure and posit a novel biomarker for IBD outcomes (402).

Despite the limited microbiological evidence, there are trends that suggest treatments for each respective disease (HEMT for CF and 5-ASA for IBD) may induce taxonomic shifts in the gut microbiota. Following initial treatment with HEMT, early results suggest partial rescue of SCFA-producing bacteria and depletion of Enterobacteriaceae members in the CF population (383, 388). Similarly, following acute treatment with 5-ASA in a UC cohort, there is an increase in select Firmicutes members and a decrease in Proteobacteria. These results, although insufficient to draw robust conclusions, suggest a progression toward microbiota recovery in these patient populations and highlight the need to develop therapies with direct effects on the dysbiotic microbiota associated with CF and IBD.

## CONCLUSIONS AND FUTURE DIRECTIONS

As we have detailed here, both IBD and CF adopt similar markers of gut microbiota dysbiosis: increased levels of Proteobacteria (largely driven by *E. coli*), decreased levels of select Firmicutes taxa (including multiple anaerobic SCFA producers), decreased Verrucomicrobiota (specifically *Akkermansia*, which is known to metabolize mucin), increased detection of oral microbes and fungi with decreases in select archaea, like methanogens. There is also decreased Bacteroidetes (*Bacteroides*) in CF, which is more nuanced in IBD microbiota studies and may be associated with the severity of disease and sample type (403). The function of the microbiota in each disease state is also shifted. In CF, metagenomic studies have reported an enrichment in fatty acid degradation microbial KEGG pathways and a depletion of fatty acid biosynthesis microbial KEGG pathways. These results, coupled with significant reductions in SCFA abundance, suggest increased breakdown and decreased production of these beneficial metabolites (34). Proteomic studies from CF stool confirm this observation through the reduction in proteins associated with butyrate production (47). In CD, there is a similar depletion of SCFAs when quantified via metabolomics. However, this depletion in SCFA is less consistently observed in UC cohorts, suggesting that more widespread inflammation, as seen in CD compared to UC, is a great contributor to the inability of commensal SCFA producers to establish or persist. Because of the small bowel disease, extensive inflammation, and consequent taxonomic and metabolic shifts observed in the intestine, we suggest that CF and CD share more commonalities in pathology compared to CF and UC.

In an attempt to separate the effects of the CFTR defect from other factors associated with CF (e.g., antibiotic use, environmental factors, microbiota, nutrition) on GI disease, Meeker et al. investigated the direct role of CF genotype (CFTR S489X) on the establishment of a gut microbiota in germ-free mice (404). Here, the authors orally gavaged non-CF and CF ex-germ-free mice with the same microbiota composition—feces from specific pathogen-free (SPF) mice—and tracked microbial composition following an establishment period of 1–3 months. This study reports significant shifts in composition between non-CF and CF mice colonized with SPF stool, as analyzed by Bray-Curtis distance. Specific taxa shifted between genotypes are concordant with what is seen in humans: a decrease in Bacteroidetes (*Parabacteroides*) and increases in *Escherichia-Shigella*, *Blautia,* and *Bifidobacterium*. These results suggest that CFTR dysregulation alone selects for a gut microbial community with features that are conserved between mice and humans (404). In addition, CF mice (both germ-free and SPF-colonized) exhibit histological pathologies of the intestine, including dilated Brunner’s glands within the proximal small intestine and retention of Paneth cell contents in the deep intestinal crypts. Mucus retention in Goblet cells in CF mice was also observed. Finally, the authors report an increase in TH17 responses in the spleen and mesenteric lymph nodes of CF mice, which agrees with the literature in human studies (405, 406). There were a few microbiological results that disagreed with human CF studies, such as enrichment of certain Firmicutes taxa in CF mice (e.g., *Anaerostipes* and *Lactobacillus*), which are usually depleted in pwCF. The authors attribute these inconsistencies to specific features of the murine GI lumen, or perhaps to the specific diets and therapies that cwCF receive (404). Therefore, it is important to consider other physiologies of the CF intestinal environment in the interplay with the gut microbiota.

**Fig 1:**
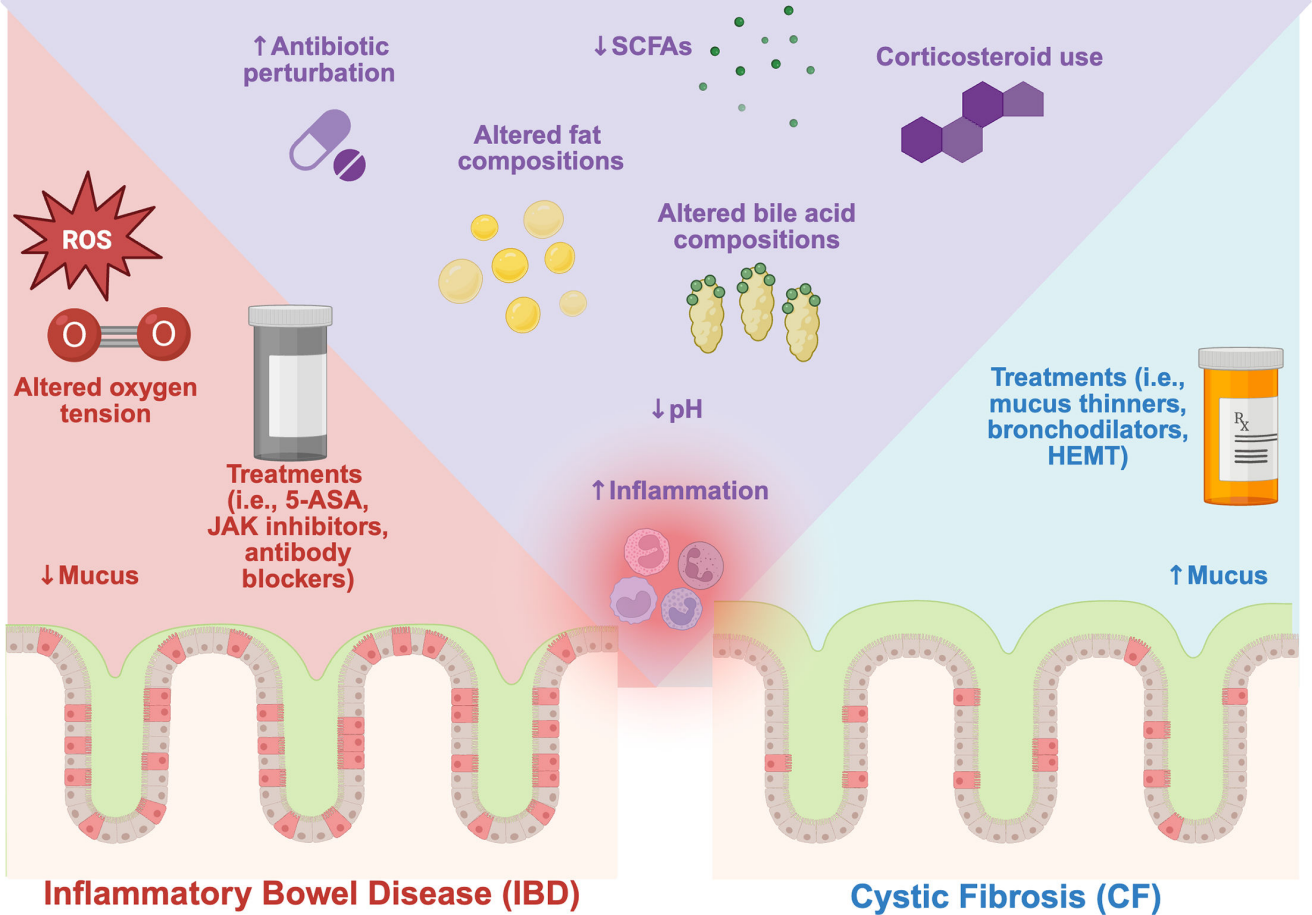
Graphical summary of the physiological alterations in IBD and CF, compared to healthy cohorts. Physiological features and alterations that are specific to the colonic environment in IBD (red, left), CF (blue, right), or shared between both diseases (purple, middle). Directional changes are relative to healthy cohorts. Figure made in Biorender.

In this review, we discuss various physiological features that are similar and different between CF and IBD intestinal environments, broadly summarized in Fig. 1. Both conditions demonstrate significant intestinal inflammation with elevated inflammatory markers, including calprotectin, S100A12, and lactoferrin. Importantly, IBD cohorts typically display increased levels of inflammation relative to CF cohorts. CF and IBD cohorts share characteristics, such as lower intestinal pH compared to healthy controls, similar bile acid dysregulation patterns, marked by increased primary bile acids and decreased secondary bile acids, and comparable alterations in fatty acid profiles, including decreased linoleic acid and increased saturated fatty acids. Both diseases enact treatment regimens commonly including antibiotics and corticosteroids. However, key differences distinguish these conditions. CF is characterized by thick, dense, highly glycosylated mucus, while IBD shows disrupted, thin mucosal layers. Disease onset timing varies significantly, with CF diagnosed in infancy while IBD typically presents in adolescence or adulthood, leading to earlier steroid and antibiotic exposure in CF patients. Treatment approaches differ substantially: CF therapy focuses on CFTR modulators and mucolytics, while IBD treatments primarily target immune responses. Inflammation patterns also differ, with CF showing consistent inflammation while IBD typically cycles through periods of active disease and remission. Treatment responses vary as well, with CF showing promising responses to CFTR modulators while IBD treatments demonstrate more variable efficacy. These similarities and differences highlight the complex nature of both conditions and underscore the unique challenges in their respective treatments, despite some shared pathological features. A key challenge is understanding which changes in intestinal physiology drive the changes in gut microbiota (and vice versa). That is, perhaps we should focus on common features of CF and IBD intestinal physiology, given the similarities in gut microbial dysbiosis for these diseases.

Canonical approaches to characterizing the gut microbiome and host physiology have used stool as a proxy for the average composition of the colon. While this sample is easily accessible and obtained through non-invasive methods as compared to a mucosal swab or luminal aspirate, stool provides a limited scope of information. Recent technologies (407) have been developed to obtain more temporal and spatial information within the GI tract by sampling different regions along the intestine for both microbial and host characteristics. Such technologies have begun to shed light on the variation that exists across regions of the GI tract at a larger scale (i.e., small intestine vs. rectum) and a smaller scale (mucosa vs. lumen). In addition, these approaches can more directly compare physiological changes that occur across diseases, potentially identifying key contributing factors (either microbial or host) that are primarily involved in shifting microbiomes, possibly despite disease etiology.

It is apparent that microbes and the physiological features of the gut are clearly interlinked. For example, certain microbes impact the metabolism of bile acids (e.g., deconjugation). On the flip side, some bile acids are detrimental to microbes (e.g., primary bile acids are typically toxic due to their detergent-like activity on membranes). Shifts in microbial composition also allude to alterations in microbial interactions and competition within the intestine. Changes in the competitive balance may purely be mediated not only by nutritional availability but also through toxic metabolic byproducts or host-targeting factors. In IBD, there is a higher detection of *pks+ E. coli*, a genotoxic strain of this microbe that synthesizes and secretes colibactin. In CF cohorts, the role of colibactin-producing strains is less defined; yet, it is hypothesized that the dysfunctional mucosal barrier in the intestine contributes to increased vulnerability to this molecule’s genotoxic activity (408). Colibactin has been implicated in colorectal cancer (CRC) risk via double-stranded DNA breaks but also altered metabolism and microbial competition. Indeed, both IBD and CF populations are at increased risk for CRC (408, 409), likely partially attributed to the involvement of colibactin. The interplay between microbe and host factor, whether it be bile acids or colibactin, is complex, with the potential for a vast spectrum of clinical outcomes. Teasing apart mechanisms and causation of a change in disease-associated physiology requires understanding of how microbes interact with their environment and host, and vice versa. At this intersection, we will be able to identify biomarkers and more effective, targeted treatments for these diseases.

Treatments targeted at microbes, such as pro- and prebiotics, have been implemented in both CF and IBD cohorts. In the context of CF, Coffey et al. summarized the results of 12 RCTs investigating the impacts of probiotic use in children and adults with CF lasting up to 12 months (410). The majority of these trials used strains of *Lactobacillus* as the probiotic (411, 412), while a few studies used a cocktail of assorted microbes (413, 414). Select RCTs have shown improvements in lung function (411) and reductions in inflammation (415, 416), decreased exacerbations (412), and overall better quality of life scores (410, 413) following relatively short durations on probiotics (i.e., 3–6 months). Studies that report outcomes up to 12 months have variable and inconclusive evidence to determine the efficacy of probiotics (417, 418). Thus, further investigation using adequately powered multicenter RCTs with longer duration times with alternative probiotic cocktail compositions is required to (i) establish the long-term impact of probiotic supplementation in CF cohorts, (ii) determine the clinical implications of the observed reduction in inflammation and lung exacerbations, and (iii) address the adverse events (i.e., vomiting, diarrhea, allergic reactions) that arise in some cases (410).

In IBD cohorts, there are comparatively more data on probiotics use than in CF cohorts, particularly as this therapy pertains to the treatment of pouchitis. Pouchitis is inflammation of the ileal pouch–anal anastomosis (IPAA) that occurs in over 50% of patients after surgery (419, 420). In this context, a probiotic cocktail, known as VSL #3, has the most robust data sets. This cocktail contains eight live bacterial strains: *Streptococcus thermophilus*, four strains of *Lactobacilli* (*L. paracasei, L. plantarum, L. acidophilus,* and *L. delbrueckii*), and three strains of *Bifidobacteria* (*B. longum, B. breve,* and *B. infantis*) (419). Its use has been shown to decrease inflammatory markers, including TNF-α, IFN-γ, and MMP-2/9, in patients with pouchitis (421). In addition, some data suggest that probiotics may be effective for the induction and maintenance of remission in mild to moderate UC, as reported through improvements in disease activity scores (422, 423) and reduced histopathology after 1 year of treatment (424). By contrast, the efficacy of probiotics in CD cohorts is less defined (425). In a randomized controlled trial of CD patients taking the same cocktail, VSL #3, there was no difference in severe lesions observed between placebo and experimental groups following 90 days of treatment (426). While pro-inflammatory cytokine levels were lower in the CD group who took probiotics, symptoms did not differ (426). Despite promising results of probiotic therapy in the prevention and treatment of pouchitis and UC, there is still no solid scientific data confirming they can significantly alter the course of the disease (427).

The variability in efficacy of probiotics highlights a key gap in knowledge: despite the numerous microbiota-disease associations, there is still no direct evidence linking any single microbe or metabolite to the etiology of IBD or CF. Therefore, it is unlikely that supplementation with a single strain, or even a small consortium of bacterial strains, will ameliorate inflammation, pathology, and/or symptomology within these diseases. Alternatively, we are not replacing key missing microbe(s) with the probiotics used to date. More recent studies have, thus, investigated the potential of fecal microbiota transplantation (FMT) in the treatment of IBD and CF. FMT is a standard treatment for the resolution of recurrent *Clostridium difficile* infections, which are frequent in both the IBD (428) and CF populations, although active disease in CF populations does not correlate with carriage of the microbe (429, 430). To the best of our knowledge, only two FMTs have been performed in pwCF, both of whom had *C. difficile* infections (429, 431). The CF patients, a male adult and a female child, were unresponsive to the canonical antibiotic therapy for *C. difficile* infections (i.e., vancomycin, metronidazole, fidaxomicin) (432) and were thus considered for FMT. The male adult also suffered from recurrent lung infections, which exacerbated the *C. difficile* infection and worsened his lung health; the FMT was considered not only to treat the intestinal infection, but also to aid in the unresponsive lung infections. In both cases, the FMT was successful in eliminating *C. difficile* infection and improving GI symptoms (429, 431). Gut microbiome analyses in the female child suggest that FMT depletes Proteobacteria (*Escherichia, Klebsiella*) and enriches for beneficial Actinobacteria (*Bifidobacterium, Collinsella*) and other SCFA producers (*Roseburia*) within 1 month post-FMT (431).

FMT as a therapy in IBD cohorts is much more heavily studied than in CF cohorts. A number of reviews have reported FMT to be effective at preventing *C. difficile* infections in patients with IBD (433–435). Many of the clinical trials assessing FMT efficacy are focused on UC cohorts, as opposed to CD cohorts. In UC, remission rates following FMT reach about 30%–40% (436–439), which is a lower success rate than seen for non-IBD patients with recurrent *C. difficile* infections. Furthermore, many of these studies followed up with patients only about 6–12 weeks following transplantation, which does not lend conclusive evidence for long-term remission rates (440). Notably, serious adverse events, such as IBD flare, hospitalization, and colectomies, have been reported post-FMT in UC cohorts, introducing some uncertainty to this therapy (438, 440). Regardless, the current evidence of FMT in inducing UC remission is promising yet incomplete due to small sample sizes in randomized controlled trials, short follow-up durations, and differences in study design (i.e., dosage, single vs. multiple FMT donors, fresh vs. frozen FMT, routes of delivery) (428, 441). Further research is needed to define a standard microbial consortium to improve safety and consistency across studies (442, 443) and/or substitute remission-associated metabolites into the treatment regimen (444–447). As of now, FMT is often delivered via colonoscopy, enema, or nasogastric tubes. It could be assumed that the use of site-directed colonization through a pH-responsive capsule, similar to the technology introduced above (407), may improve the establishment of select microorganisms in specific intestinal regions. This approach has yet to be explored.

As discussed here, we directly compare the microbiota and intestinal environments across CF and IBD cohorts and show some key similarities and differences across these diseases. It is evident that both diseases are multifaceted and result from either genetic predisposition or susceptibility, environmental conditions, and a gut microbiota with signatures of dysbiosis. To disentangle these contributing factors and properly determine the role of each, it is crucial to fully understand the interaction between the host, the environment, and the microbiota. Indeed, the lack of such understanding is reflected in the lack of effective treatments in these populations. The complexity of understanding the various drivers of the shifting microbiota is immense; through using human and animal models, it is extremely difficult to dissect whether changes in host shift the microbiota, or whether microbial dysbiosis negatively impacts the host (or both). The analyses presented here provide important information that could be used to develop *in vitro* models to better understand the factors driving dysbiosis. For example, a recent study from our team reported a medium called CF-MiPro that could maintain CF gut microbiota *in vitro* or shift a normal microbiota to a CF-like community (46). CF-MiPro is based on a previously reported MiPro medium (448), but modified to reflect a CF-like gut environment, with a reduced pH, increased mucin, fat, reactive oxygen species, and bile, and the presence of metabolites like nitrate, sulfate, and formate associated with inflammation. In principle, this medium could be used to dissect specific factors driving microbial dysbiosis. Importantly, while an *in vitro* medium model lacks host factors, it can include features that reflect the host-derived factors outlined above. Furthermore, the medium composition can be altered to reflect specific diseases (i.e., more mucin in CF, less in IBD). Alternatively, model systems that incorporate host features, such as organoids (449) and/or gut-on-a-chip models (450), may be implemented to dissect the microbiological and physiological alterations that occur in a controlled setting without excluding host contributions. The observed similarities in gut microbiota structure across the diseases discussed in this review suggest an interesting avenue of exploration through the use of synthetic microbial communities (451). Questions to probe may include the following. How does a conserved community respond and/or adapt to the CF intestinal environment versus the CD/UC intestinal environment? Which taxa are largely contributing to metabolic changes under each respective condition? In the context of a community, which disease-associated physiological feature(s) drive the largest ecological shifts? In conclusion, the information presented here sets the framework for building models to move beyond observational studies to understand the mechanisms that drive dysbiosis for diseases like CF and IBD.
